# From Neonatal Intensive Care to Neurocritical Care: Is It Still a Mirage? The Sicilian Multicenter Project

**DOI:** 10.1155/2021/1782406

**Published:** 2021-08-13

**Authors:** Raffaele Falsaperla, Laura Mauceri, Milena Motta, Ettore Piro, Gabriella D'Angelo, Eloisa Gitto, Giovanni Corsello, Martino Ruggieri

**Affiliations:** ^1^Neonatal Intensive Care Unit, AUO Policlinico “Rodolico-San Marco”, University of Catania, Catania, Italy; ^2^General Pediatrics, Acute End Emergency Pediatric Unit, AUO Policlinico “Rodolico-San Marco”, University of Catania, Catania, Italy; ^3^Neonatal Intensive Care Unit, University Hospital “P. Giaccone”, Department of Sciences for Health Promotion, Maternal Infant Care, Internal Medicine and Medical Specialties “G. D'Alessandro”, Neonatal Intensive Care Unit, Via A. Giordano 3, 90127 Palermo, Italy; ^4^Neonatal Intensive Care Unit, Department of Pediatrics, University of Messina, Messina, Italy; ^5^Unit of Pediatrics and Pediatric Emergency, AUO Policlinico “Rodolico-San Marco”, Department of Clinical and Experimental Medicine Section of Pediatrics and Child Neuropsychiatry, AUO Policlinico Vittorio Emanuele, University of Catania, Catania, Italy

## Abstract

**Background:**

Neonatal brain injury (NBI) can lead to a significant neurological disability or even death. After decades of intense efforts to improve neonatal intensive care and survival of critically ill newborns, the focus today is an improved long-term neurological outcome through brain-focused care. The goal of neuroprotection in the neonatal intensive care unit (NICU) is the prevention of new or worsening NBI in premature and term newborns. As a result, the neonatal neurocritical care unit (NNCU) has been emerging as a model of care to decrease NBI and improve the long-term neurodevelopment in critically ill neonates.

**Purpose:**

Neurocritical care (NCC) Sicilian project includes three academic sites with NICU in Sicily (Catania, Messina, and Palermo), and its primary goal is to develop neurocritical neonatal care unit (NNCU).

**Methods:**

In 2018, the three NICUs created a dedicated space for neonates with primary neurological diagnosis or at risk for neurological injuries—NNCU. Admission criteria for eligible patients and treatment protocols were created. Contact with parents, environmental protection, basic monitoring, brain monitoring, pharmacological therapy, and organization of the staff were protocolized.

**Results:**

Evaluation of the efforts to establish NNCU within existing NICU, current protocols, and encountered problems are shown. Implications for Practice. Our outcome confirmed the need for dedicated NNCU for neuroprotection of critically ill neonates at risk for a neurological injury. Although the literature on neonatal neurocritical care is still scarce, we see the value of such targeted approach to newborn brain protection and therefore we will continue developing our NNCU, even though there have been problems encountered. The project of building NNCU will continue to be closely monitored.

**Conclusions:**

The development of our neonatal neurocritical model of care is far from being completed. Although it is currently limited to the Sicilian area only, the goal of this paper is to share the development of this multicenter interdisciplinary project focused on a newborn brain protection. After evaluating our outcome, we strongly believe that a combined expertise in neonatal neurology and neonatal critical care can lead to an improved neurodevelopmental outcome for critically ill neonates, from the extremely preterm to those with brain injuries.

## 1. Introduction

Neonatal brain injury (NBI) can lead to death or significant neurological impairment, often associated with significant economic and social impact [1]. The risk of learning disabilities and cognitive and neurological impairment increases with a decreasing gestational age [2]. Approximately 50% of preterm infants will be left with various degrees of cognitive, behavioral, and social impairments.

After many years of intense efforts to improve the delivery of the neonatal intensive care and increase survival of critically ill newborns, the main focus today is to improve long-term neurological outcome [3]. Despite the improved survival of extremely preterm neonates over the last decade, the incidence of neurodevelopmental impairment (NDI) has not decreased substantially [4]. In adult medicine, neurocritical care (NCC) is a well-established model of care leading to an improved outcome [5, 6]. Brain-focused care has been only more recently applied to critically ill children and neonates [3, 7–9]. The main goal of NCC is the neuroprotection and prevention of NBI in premature and term newborns at risk for adverse neurological outcome. The recent literature supports the neurocritical model of care to decrease the NDI and to improve short-term and long-term neurodevelopmental outcome [10, 11] in neonates at risk.

The neonatal neurocritical model of care aims to improve neurodevelopmental outcomes for high-risk infants by having a dedicated neonatal neurologist and using advanced brain neuromonitoring techniques for timely detection and characterization of neurological injuries [12].

The NCC Sicilian project includes three academic cities with NICUs (Catania, Messina, and Palermo). The goal of this project was to create a neonatal neurological care unit (NNCU) at each site comprising a multidisciplinary team which includes neurologists, neuroradiologists, and developmental specialists with a goal to improve neuroprotective care for neonates at risk for adverse neurological outcome [13].

**Figure 1:**
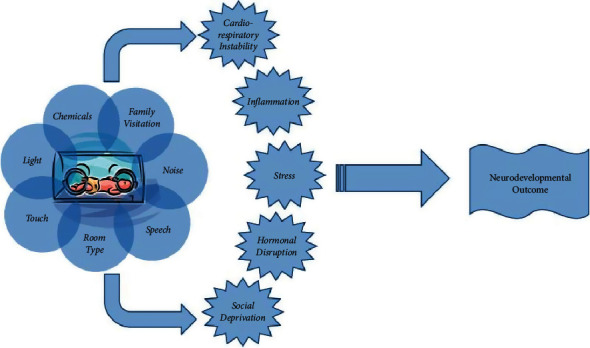
The environmental factors that contribute to the development of a newborn during hospitalization in the NICU are numerous and occur in a critical period for neurodevelopment (adapted from Santos et al. [17]).

Using the most current scientific evidence, our goals included contact with family [14, 15], environmental protection (Figure 1) [16, 17], improvement in quality of sleep in critically ill newborns [18, 19], multimodal basic monitoring and neuromonitoring [20, 21], pharmacological neuroprotection [22], and the development of NNCU consisting of trained neonatologists and specialized nurses [23] undergoing continuing education and regular training courses.

We are here presenting our results one year after NNCU project initiation.

## 2. Materials and Methods

### 2.1. Methods

In 2018, the three Sicilian NICUs created a dedicated space, NNCU, for neonates with primary neurological diagnosis as well as for those at risk for brain injuries. Specific diagnoses including extreme prematurity were included in the eligibility criteria for NNCU admission. Protocols for neuromonitoring and when to consult neurology or neurosurgery services were developed. The admission to NNCU was decided either at birth for inborn neonates, at hospital admission for outborn neonates, or upon the identification by the neonatal team (attending neonatologist, charge nurse, and the neonatal fellow) of an emerging “at-risk profile” or neurological problem (Table 1). The goal was an early identification of newborns at risk who could benefit from neuroprotective care, specifically neuromonitoring techniques, enhanced brain-focused nursing care, and specific therapeutical approaches.

### 2.2. Setting and Population

The University San Marco Hospital of Catania has a delivery unit with approximately 2000 deliveries in a year. The NICU is also a reference center for the neonatal emergency transport service (NETS). In Catania NICU, the incidence of prematurity is approximately 1 preterm/8 newborns, an incidence of HIE is about 0.3% of all hospitalized neonates, and an incidence of perinatal stroke is about 0.1% hospitalized neonates.

The NICU at the University Hospital of Palermo has a delivery unit with approximately 900 deliveries in a year. It serves as a referral center for neonates requiring surgery and newborns with genetic diseases, malformations, and metabolic diseases in western Sicily. Preterm births account for 11%, and HIE (stages II and III) and neonatal stroke encompass 0.4% and 0.3% of newborns, respectively.

The NICU at the University Hospital of Messina is associated with a delivery center with 1340 deliveries in a year. It is a referral center for NETS and for newborns with genetic diseases, malformations, and metabolic diseases. Preterm births account for about 5.3% and HIE for about 0.40% (Table 2).

Prior to the project initiation, all three NNCUs developed a series of shared organizational steps and set the goals to be reached within a period of 12 months.  Table 3 summarizes predetermined goals in all three Sicilian NNCUs.

### 2.3. Education and Implementation

The first step in the development of our NNCUs was the encouragement of the parent-infant bonding time [24]. Our ultimate goal was to have neuro-NICU available to the parents for 24 hours, but as the first step, we aimed to arrange for a comfortable overnight stay for the mother. Our goal was to organize real-time video monitoring in the NNCU for the parents who were not able to visit their newborn. To minimize parental stress and to maximize parental involvement, the care team was responsible for providing frequent updates on the treatment plan and interventions for their newborns in the NNCU.

The second fundamental step in the development of the NNCU was to increase the environmental protection. To protect neonates from unnecessary light, we have reduced light stimulations, aiming to use spotlights for procedures and keep the lights dim. Dark curtains over the incubators were used for newborn protection, and only indirect ambient lighting was available in the room. To protect neonates from noise, we reduced acoustic stimuli, which included elimination of loud voices near the incubators, silencing the alarms as soon as possible, and avoiding loud equipment near the incubators. We had set up luminous alarms calibrated on a decibel threshold. To protect the newborns from excessive tactile stimuli, all the neuro-NICU staff were instructed to focus on gentle handling when carrying out the daily routine care. We reduced painful procedures to absolute minimum required for optimal care. We also included sleep in the environmental protection. Clinicians are often unable to reliably assess a newborn's sleep cycle and therefore they cannot give routine time care and interventions such as feeding or therapy according to the sleep-wake cycle. We aim to adopt a reliable sleep assessment system through the use of neuromonitoring technologies. Currently, we plan to monitor the sleep cycle in critically ill neonates using a continuous video EEG polygraphy.

### 2.4. Assessment and Measures

The third fundamental step was a continuous vital sign monitoring, including heart rate, blood pressure, peripheral oxygen saturation, and temperature. Glucose levels were monitored with the goal to maintain normoglycemia, a key step in prevention of the secondary brain injury. In the ventilated newborns, we aimed for normocarbia/permissive mild hypercapnia and to avoid hypoxemia. The plan moving forward is a transcutaneous measurement of oxygen (TcPO_2_) and carbon dioxide (TcPCO_2_).

The fourth step included neuromonitoring, using aEEG and cEEG, and neuroimaging.

A continuous video EEG polygraphy is the gold standard for seizure detection in neonates. However, if continuous EEG is not available, a prolonged monitoring using a neonatal montage with 8 brain derivations, eye leads, ECG lead, respiratory lead, and electromyography (EMG) lead with concomitant video recording can also be used for seizure detection and background evaluation. If conventional EEG is not available, amplitude-integrated EEG (aEEG) has been shown to be better than no monitoring.

Bedside cerebral ultrasonography and an access to brain MRI are available in all three NNCUs.

Visual and auditory evoked potentials are used in the term and preterm newborns for monitoring of visual and auditory pathway maturation, and they are especially useful in the context of posthemorrhagic ventricular dilatation in preterm neonates, hypoxic ischemic encephalopathy, stroke, cerebral malformation, and hydrocephalus [25].

The fifth step in the development of our NNCU is pharmacological neuroprotection [22]. Therapeutic hypothermia is a standard of care in all 3 neurocritical NICUs. Our first-line anticonvulsant is levetiracetam (LEV) rather than phenobarbital, considering the potential neuroprotective effects of LEV.

As the evidence from building other neurocritical NICUs suggested [26], we identified the NNCU team. Ideally, one nurse with NNCU training is always available for a newborn admitted to NNCU. At least three neonatologists with training in neurocritical NICU are part of the team at each site. NNCU director position is shared between a neonatologist and neonatal neurologist, and one of them is always available [23]. A neurophysiology technician is available around the clock.

Furthermore, advanced education in the form of a two-day training course was organized by the NNCU staff at the universities of Catania, Palermo, and Messina. The goal of the course was to advance participants' knowledge on the key neonatal neurology topics and to implement and share neonatal neurocritical care program protocols. The presentations included fetal and neonatal brain development, CNS malformations, HIE, role of therapeutic hypothermia, newborn neurological examination, Sarnat evaluation, neuroprotective care at the bedside, neonatal neuroimaging (brain Doppler, cerebral US, and MRI), brain monitoring using aEEG, video EEG polygraphy, seizure detection and seizure management, palliative care, and follow-up program for high-risk infants. The course also included a session with familial experience.

### 2.5. Analysis

The goal of the NNCU development project was to introduce a highly specialized brain-focused care for newborns with or at risk of brain injury. The secondary goal was to reduce newborn mortality and improve psychomotor outcome in high-risk newborns by employing neurocritical NICU care.

## 3. Results

The results shown represent a one-year point in the evaluation since the project of developing the neurocritical model of care in NICU started. We are presenting the problems encountered and the ongoing projects (Table 3):

  Step 1: all 3 NNCUs have been open to parents for 12 hours daily. We noticed that, at home, childcare issues, work commitments, and distance from home were often the reasons parents were unable to spend time at the bedside. Therefore, NNCU staff connected with the families using FaceTime, Skype, or similar applications. Parents received support and additional education as needed while they were preparing to deal with short- and long-term challenges of caring for an infant with potentially adverse neurodevelopmental outcome. Frequent updates about the treatment plan and interventions were also provided by the NNCU team over the phone as needed.
  Step 2: to achieve our goal of environmental protection, light stimulations were reduced in each of the three neurocritical NICUs. Spotlights were used for procedures, while the lights in the unit were kept dim. Furthermore, dark curtains were placed over the incubators for further newborn protection. Acoustic stimuli were also reduced by eliminating high-volume care team discussions and by avoiding the closeness of the noisy equipment near the incubators.

In one NNCU, we detected problems with acoustic alarms due to the excessive sensitivity of the cardiopulmonary monitors and the inability to calibrate their intensity to the decibel threshold.

All neurocritical NICU staff received adequate training on how to handle emergency carts, ultrasound scanners, and scales without excessive noise and how to slowly open/close refrigerators, drawers, and cabinets. They were also trained on how to minimize unnecessary tactile stimuli and how to eliminate or at least reduce painful procedures.

To improve sleep, one of the NNCUs attempted to use a continuous video EEG monitoring to avoid sleep cycle interruption. However, this turned out to be too difficult to perform for all at-risk neonates, and it also interfered with goal of minimum handling and reducing tactile stimulation. Therefore, at the end, in all three NNCUs, the medical and nursing staff used their clinical judgment and clinical skills to minimize sleep interruptions by feeding, care, or therapies.

In all three NNCUs, vital signs including heart rate, blood pressure, peripheral oxygen saturation, and temperature were continuously measured continuously. Blood glucose levels were also monitored to maintain normoglycemia.

In all three NNCUs, neuromonitoring using aEEG was a standard of care in all neonates with HIE undergoing therapeutic hypothermia. Video EEG polygraphy was used on case-by-case basis. In one NNCU site, a continuous video EEG polygraphy using a transversal montage with 8 brain derivations, eye movement electrodes, ECG, respiratory monitoring, and EMG leads was initiated for every neonate with neurological diagnosis on admission and repeated as required clinically. In the other two NNCUs, conventional EEG was performed if there was a suspicion for seizures on the aEEG or clinically. In two out of three NNCUs, a neurophysiology technologist was available during weekday working hours only.

At the time of the project initiation, near-infrared spectroscopy (NIRS) was routinely used in two NNCUs, and in one NNCU, it became part of routine care during the last two months of the study period.

Visual and brainstem evoked potentials were routinely performed only in one NNCU. Cerebral ultrasonography and Doppler ultrasound were performed in all NNCUs on admission. Follow-up ultrasound during the admission was based on the clinical judgment and care protocols. Brain MRI was also done as per individual care protocols or as per attendee's request.

Therapeutic hypothermia was used as a standard of care to all newborns at 35 weeks of gestation or older who suffered a hypoxic-ischemic event and met the established criteria. Seizures were treated with anticonvulsants. LEV was preferred to phenobarbital for its neuroprotective effects and initiated at standard doses (initially, 10 mg/kg divided twice daily, with a gradual increase to up to 40 mg/kg, divided twice daily in case of no response to lower doses). Phenobarbital was used as adjunctive therapy in those cases resistant to LEV [27].

To prevent and treat apneic spells in the preterm newborns, caffeine was administered at 20 mg/kg dose and a maintenance dose of 5 mg/kg/daily [28].

In one of the NNCU, a double-blind study using melatonin in neonates with HIE is currently ongoing [29].

In all three NNCUs, one of the critical issues was lack of trained healthcare workers. Despite the administrative issues, the interest in the project motivated the NICU staff to adhere to the organizational protocols and procedures to provide advanced neurocritical care. The annual residential training course had to be put on hold due to the COVID-19 pandemic.

## 4. Discussion

Evidence for brain-focused care in critically ill neonates is growing. Therefore, three academic NICUs in Sicily started a pilot project on the development of neurocritical NICU model of care [30]. We created a dedicated space for neonates requiring NNCU level of care. The goal was to prevent NBI and to provide optimal brain-focused care to neonates with primary or secondary neurological diagnosis by staff with advanced neurocritical training and by using well-defined diagnostic and therapeutical pathways [31].

We are presenting the results of the first year of such dedicated care. We encountered many difficulties, and the NNCU development is far from being complete. Nevertheless, we are committed to the project in order to improve outcome for neonates at risk for adverse neurological or neurodevelopmental outcomes. The approach to the critically ill newborns with, or at risk of, NBI is complex and requires a multidisciplinary team.

With the recent advancements in neonatology and neonatal neurology and an increasing focus on neuroprotection in NICU, prevention of new or worsening brain injury in at-risk newborns has become a goal of an intense clinical and preclinical research, together with better diagnostic and therapeutic options for this population.

Early parent-infant bonding and skin-to-skin contact are very important for the child's neurodevelopment and the well-being of the entire family. Research suggests that lack of interaction between the mother and infant in the early postnatal period can lead to an abnormal emotional and cognitive development [14, 15, 32].

Environmental protection was another key step in the development of our NCCU, and this is applied to both term and preterm neonates (Figure 1) [17]. The earlier gestational age at birth leads to the more vulnerability to brain injuries and potentially adverse neurodevelopmental outcomes. Therefore, evidence-based and consistent brain-focused care is the key to neonatal neuroprotection, and this includes sleep. As reported by Rioulaen and colleagues, during late fetal and early neonatal stages of human development, sleeping is the primary activity of the brain [19]. In particular, the importance of REM sleep for fetal and neonatal brain maturation has been suggested in preclinical studies [33]. Even in neonates, the American Academy of Sleep Medicine suggested to differentiate stages of the sleep-wake cycle: wakefulness (*W*), wakefulness/sleep transitions, non-rapid eye movement (NREM) sleep (quiet sleep), rapid eye movement (REM) sleep (active sleep), and transitional sleep (*T*) (indeterminate sleep) [34]. There are many reasons why sleep can be interrupted in a busy intensive care unit.

An important part of brain-focused care in critically ill neonates is the assessment of their brain function. The American Clinical Neurophysiology Society recommends continuous EEG (cEEG) monitoring for all critically ill neonates; however, if full-montage continuous EEG is not available, amplitude-integrated EEG (aEEG) is considered better that no monitoring [21].

Continuous neuromonitoring can assess the presence of sleep-wake cycle and evaluate background activity and presence of seizures as shown by Ballabi in 2014 [20]. Clinical detection of seizures in critically ill neonates is insufficient, since subtle seizures and electrographic-only seizures are very common in this population. There is now enough evidence that not only electroclinical seizures but also electrographic-only seizures can contribute to worse neurological outcome in neonates with brain injuries [35]. Therefore, early seizure detection and timely seizure treatment have a potential to improve outcome [26, 36]. Pharmacological neuroprotection in NNCU includes rapid and adequate seizure treatment [37]. Seizures in neonates should be treated early. Our preference is levetiracetam as a first-line medication due to its neuroprotective effects, as opposed to phenobarbital, as we have shown previously [38].

In all three NNCUs, all neonates with hypoxic-ischemic encephalopathy that meet cooling criteria undergo therapeutic hypothermia, the only evidence-based treatment that can improve outcome after perinatal asphyxia [22].

Neuroimaging is available in all three NNCUs. A useful noninvasive bedside tool for diagnosis of structural abnormalities and brain injuries is cerebral ultrasonography [39, 40], available in all three centers. However, to fully assess the extent of brain injuries, access to brain MRI is an important part of building NNCU [41].

The importance of a dedicated neonatal neurologist in the NNCU team was suggested by Glass et al. [23, 42]. The neurologist should be involved in the care of all NNCUs patients from admission to discharge. We found that nurses with specialized training in brain-focused care are also necessary for bedside care [43]. Therefore, we developed regular training courses for NNCU staff, physicians, and nurses, as suggested by Darh et al. in 2017 [44].

Our first year of NNCU project was met with many difficulties. Specialized neonatal neurocritical care for neonates at risk for brain injury is still a relatively new subspecialty combining expertise in neurology and neonatal critical care medicine [45]. While there is strong evidence that adults with acute neurological conditions benefit from a specialized neurocritical care, the impact of such care in the neonatal population is less clear [8, 46]. However, emerging evidence suggests its potentially positive impact on neurodevelopmental outcome [24, 31, 46–48]. Glass et al. demonstrated that the rate of epilepsy after acute symptomatic neonatal seizures was lower in a cohort of newborns managed by a neonatal neurocritical care service [10].

## 5. Conclusion

Neonatal neurocritical care is a relatively new concept in the area of neonatal intensive care, unlike in the adult care. The evidence of its impact is gradually emerging. Many issues remain to be solved one year since the project initiation; however, from our three NNCUs in Catania, we are convinced that dedicated brain-focused care for critically ill neonates has a potential to improve the neurodevelopmental outcome in this population. We have developed continuing education plans for the NNCU staff. Prospective studies are needed to investigate the utility of neurocritical neonatal care and the impact on acute care and long-term neurodevelopmental outcomes.

## Figures and Tables

**Table 1:** Neonatal neurocritical care populations modified from Glass et al.'s study in *Seminars inPediatric Neurology* [9].

Acute acquired brain injury	Seizures	High risk for acquired brain injury	Developmental anomalies
Hypoxic-ischemic encephalopathy (HIE)	Acute symptomatic seizures	Encephalopathy	Brain malformation
Arterial and venous ischemic stroke	Neonatal onset epilepsies (benign and malignant)	Extremely low gestational age (<28 weeks of gestation at birth)	Microcephaly
Intracranial parenchymal hemorrhage		Hydrocephalus	Dysmorphic neonate
High-grade intraventricular hemorrhage		Need for extracorporeal membrane oxygenation (ECMO)	Multiple congenital anomalies
Meningoencephalitis		Congenital heart malformations	
Inborn error of metabolism		Postnatal cardiopulmonary arrest	
		Vascular malformations of the central nervous system	
		Symptomatic hypoglycemia	

**Table 2:** The number of births/year and the incidence of cases in our three Sicilian centers.

	Number of newborns/year	Preterm incidence (%)	HIE incidence (%)	Perinatal stroke incidence^*∗*^ (%)
Catania	2000	12.5	0.3	0.1
Palermo	700	10.8	0.4	0.3
Messina	1341	5.3	0.37	0

^*∗*^Arterial ischemic stroke, hemorrhagic stroke (IVH excluded), cerebral sinovenous thrombosis, and periventricular venous infarction.

**Table 3:** The objectives set and those achieved in our three Sicilian centers.

Prefixed goal	Contact with parents 24/24 h	Environmental protection Light, sound, touch	Basic physiology monitoring	Brain monitoring aEEG, cEEG, videopolygraphy, CUS, NIRS, evoked potentials	Neuroprotection Hypothermia and pharmacological agents	Promoting sleep Sleep monitoring with videopolygraphy	Dedicated staff 5 neuronurses, 3 neuroneonatologists, 1 supervisor, 1 neurophysiology technician	Training courses Two-day courses every 6 months
NICU 1 reached result (after 1 year)	12/24 h	Yes	Yes	aEEG, cEEG, videopolygraphy, CUS, NIRS	Hypothermia LEV, PB, caffeine	Only clinical observation	3 neuronurses, 3 neuroneonatologists, 1 neurophysiology technician (only daily for five days)	One course at beginning
NICU reached result (after 1 year)	12/24 h	Yes	Yes	aEEG, cEEG, CUS, evoked potentials	Hypothermia LEV, PB, caffeine	Only clinical observation	2 neuronurses, 1 neuroneonatologist	One course at beginning
NICU 3 reached result (after 1 year)	12/24 h	Yes	Yes	aEEG, cEEG (only selected cases), CUS, NIRS	Hypothermia LEV, PB, caffeine, melatonin	Only clinical observation	2 neuronurses, 1 neuroneonatologist, 1 neurophysiology technician (only daily for five days)	One course at beginning

## Data Availability

The data used to support the findings of this study are included within the article.
